# A complex pheotype in a girl with a novel heterozygous missense variant (*p.Ile56Phe*) of the *GNAS* gene

**DOI:** 10.1186/s13023-022-02252-6

**Published:** 2022-02-23

**Authors:** Cavarzere Paolo, Gastaldi Andrea, Francesca Marta Elli, Gaudino Rossella, Peverelli Erika, Brugnara Milena, Susanne Thiele, Granata Francesca, Mantovani Giovanna, Antoniazzi Franco

**Affiliations:** 1 Pediatric Division, Department of Pediatrics, University Hospital of Verona, Piazzale Stefani 1, 37126 Verona, Italy; 2 Endocrinology Unit, Department of Clinical Sciences and Community Health, University of Milan, Fondazione IRCCS Ca’ Granda Ospedale Maggiore Policlinico, Milan, Italy; 3 Pediatric Clinic, Department Surgical Sciences, Dentistry, Gynecology and Pediatrics, University of Verona, Verona, Italy; 4 Division of Paediatric Endocrinology and Diabetes, Department of Pediatrics, University of Lübeck, Luebeck, Germany; 5 General Medicine Unit, Fondazione IRCCS Ca’ Granda Ospedale Maggiore Policlinico, Milan, Italy; 6 Regional Center for the Diagnosis and Treatment of Children and Adolescents Rare Skeletal Disorders. Pediatric Clinic, Department of Surgical Sciences, Dentistry, Gynecology and Pediatrics, University of Verona, Verona, Italy

**Keywords:** *GNAS* gene, Hyponatremia, Subclinical hyperthyroidism, Precocious thelarche, Congenital bone abnormalities

## Abstract

**Background:**

*GNAS* is a complex gene that encodes Gsα, a signaling protein that triggers a complex network of pathways. Heterozygous inactivating mutations in Gsα-coding *GNAS* exons cause hormonal resistance; on the contrary, activating mutations in Gsα result in constitutive cAMP stimulation. Recent research has described a clinical condition characterized by both gain and loss of Gsα function, due to a heterozygous de novo variant of the maternal *GNAS* allele.

**Patients and methods:**

We describe a girl with a complex combination of clinical signs and a new heterozygous *GNAS* variant. For the molecular analysis of *GNAS* gene, DNA samples of the proband and her parents were extracted from their peripheral blood samples. In silico analysis was performed to predict the possible in vivo effect of the detected novel genetic variant. The activity of Gsα protein was in vitro analyzed from samples of erythrocyte membranes, recovered from heparinized blood samples.

**Results:**

We found a new heterozygous missense c.166A > T—(p.Ile56Phe) *GNAS* variant in exon 2, inherited from the mother that determined a reduced activity of 50% of Gsα protein function. The analysis of her parents showed a 20–25% reduction in Gsα protein activity in the mother and a normal function in the father. Clinically our patient presented a multisystemic disorder characterized by hyponatremia compatible with a nephrogenic syndrome of inappropriate antidiuresis, subclinical hyperthyroidism, subclinical hypercortisolism, precocious thelarche and pubarche and congenital bone abnormalities.

**Conclusions:**

This is the first time that the new variant c.166A > T (p.Ile56Phe) on exon 2 of *GNAS* gene, originated on maternal allele, has been described as probable cause of a multisystemic disorder. Although the mutation is associated with a reduced activity of the function of Gsα protein, this unusual phenotype on the contrary suggests a mild functional gain.

## Introduction

Guanine Nucleotide-binding protein, Alpha Stimulating (*GNAS*) is a complex gene locus located on chromosome 20q13.11 [1, 2]. It encodes multiple gene products, including the best-known alpha-subunit of the stimulatory guanine nucleotide-binding protein (Gsα), a signaling protein that triggers a complex network of pathways necessary for the actions of several hormones, neurotransmitters, and autocrine/paracrine factors [3, 4]. Gsα protein exists in a GDP-bound form at the basal state as part of a heterotrimeric complex. Agonist-activation of a Gsα-coupled heptahelical transmembrane receptor induces a GDP-GTP exchange, leading to the dissociation of Gsα from Gβγ subunits. The best-known effect is the stimulation of adenylyl cyclase and subsequent conversion of ATP into the second messenger cAMP [5, 6]. Hormonal pathways mediated by Gsα signal transduction include: parathyroid hormone 1 receptor (PTH1R), thyroid-stimulating hormone receptor (TSHR), follicle-stimulating hormone receptor (FSHR), luteinizing hormone/choriogonadotropin receptor (LHCGR), growth hormone–releasing hormone receptor (GHRHR), melanocortin 2 receptor (MC2R) and arginine vasopressin receptor 2 (AVPR2) [7–9].

Heterozygous inactivating mutations in Gsα-coding *GNAS* exons can cause different phenotypic expressions that vary from pseudohypoparathyroidism (PHP) to pseudo-pseudohypoparathyroidism (PPHP) to Progressive Osseous Heteroplasia (POH) [3, 10]. On the contrary, activating mutations in Gsα result in constitutive cAMP signaling due to inhibition of intrinsic GTP hydrolase activity, leading to different clinical phenotypes as fibrous dysplasia or McCune-Albright Syndrome (MAS) [3, 11–13]. Recently, a new de novo variant on maternal *GNAS* allele has been described as causative of a clinical condition characterized by both gain and loss of Gsα function [14], although the first case of gain and loss of function in a patient with a Gsα mutation dates back to 1994 [15]. *GNAS* expression is regulated both by tissue-specific imprinting and alternative splicing. Parental dependent epigenetic mechanisms are involved in gene regulation, with predominantly maternal expression in specific tissues [3, 4, 16, 17].

We here report a girl with a complex combination of apparently unrelated clinical signs: persistent and severe hyponatremia, subclinical hyperthyroidism, precocious thelarche and pubarche and congenital bone abnormalities. This unusual phenotype characterized by symptoms that might suggest a gain of function was unexpectedly explained by a new heterozygous missense c.166A > T—(p.Ile56Phe) *GNAS* variant that determined a reduced activity of 50% of Gsα protein function.

## Patient

**Fig. 1:**
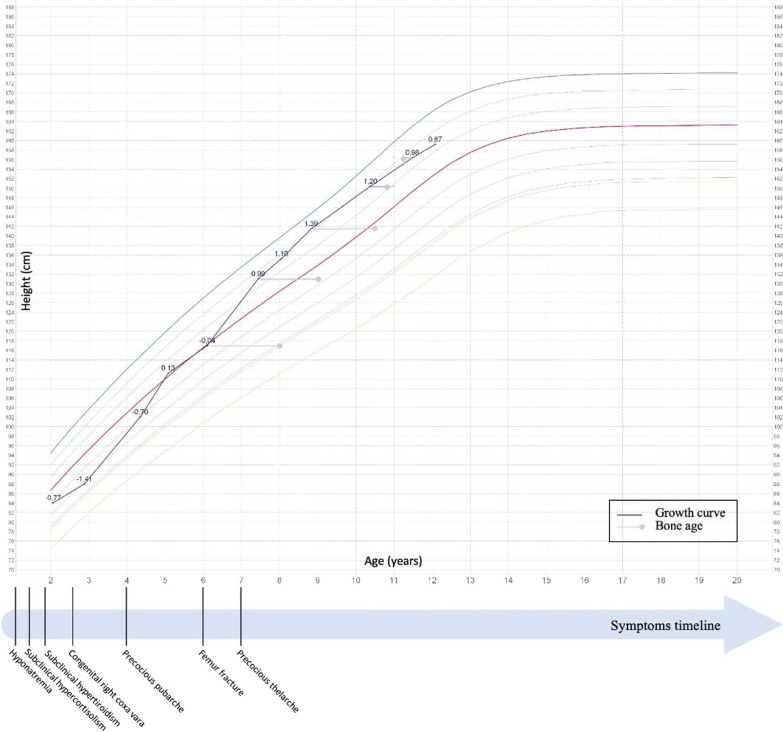
Growth chart of our patient. We also report the different symptoms in chronological order of appearance

The following description focuses on the clinical history of a female infant who is today 14 years old and presents a weight of 55.0 kg (0.20 SDS) a height of 165.7 cm (1.02 SDS) and a BMI of 20.0 kg/m^2^ (-0.20 SDS). From birth, she displayed different clinical signs and symptoms that seemed independent from each other, at first glance, but during infanthood assumed a more precise clinical significance allowing us to establish a diagnosis. In the next sections, we will report the different symptoms in chronological order of appearance (Fig. 1).

### Hyponatremia

She was born at term (gestational age of 38 weeks) by vaginal delivery after an uneventful pregnancy. Parents are not related. No noteworthy diseases were reported in her family history, the mother in particular showed no phenotypic signs and presented normal hormonal levels. Birth weight was 3410 g (0.28 SDS), birth length 52 cm (1.44 SDS); Apgar score: 8 at first minute of life and 10 at fifth minute of life. Her newborn screening for congenital adrenal hyperplasia (CAH) resulted positive, so she was submitted to clinical, biochemical and radiological examinations to confirm the diagnosis. These exams evidenced: Na 127 mmol/L (normal range 135–145 nmol/L), K 4.9 mmol/L (normal range 3.4–4.8 nmol/L), Cl 94 mmol/L (normal range 94–106 nmol/L), 17-OHP 244.8 nmol/L (normal range for age: < 10 nmol/L); ultrasound investigation showed the presence of bilateral hypertrophy of adrenal glands. Nevertheless, her general conditions were good with complete absence of signs of virilization and a regular growth at breastfeeding. With the suspicion of CAH, the clinical team of the hospital where she was born treated her with cortisone acetate 25 mg/m^2^, NaCl, later adding fludrocortisone. Despite the treatment, Na concentrations remained low (between 119 and 128 mmol/L) in absence of hyperkalemia, while 17-OHP levels reverted to normal range (6.0 nmol/L).

At 6 months of life, she came for the first time to our attention. She presented good general conditions: euvolemic, with normal peripheral perfusion, normal external genitalia without virilization signs, with slight systolic hypertension for age (107/56 mmHg). Blood exam showed Na 128 nmol/L, ACTH < 5 pg/mL (normal range 8–59 pg/mL), cortisol at 8:00 AM 20 µg/dL (normal range 4–17 µg/dL). At abdominal ultrasonography, adrenal glands appeared normal. According to these data, we gradually reduced cortisone acetate and fludrocortisone doses and increased sodium chloride supplementation up to 8 mEq/kg/day with normalization of Na values. At 7 months of life cortisone treatment was definitively stopped. Adrenal function remained normal with cortisol concentrations ranging between 13.6 and 15.5 µg/dL, but ACTH level was still unmeasurable. ACTH stimulation test ruled out a central adrenal insufficiency. Initially we hypothesized that low ACTH level resulted from a long-term steroid use but the persistence of this data over time has allowed to define a condition of subclinical hypercortisolism. As she persisted in euvolemic state with normal peripheral perfusion and continued to present sporadic and asymptomatic episodes of hyponatremia despite the supplementation with NaCl, she was admitted again to our department. During this second hospitalization period, we investigated more in detail the cause of her hyponatriemia. We ruled out renal tubulopathy (normal urinary electrolytes and phosphate excretion, normal acid–base balance) and cystic fibrosis (the sweat test resulted negative). We confirmed low serum Na levels associated with low plasma osmolarity (262 mmol/kg) and inappropriately elevated urinary osmolarity (832 mmol/Kg). ADH level was in the normal range (between 4.26 and 5.85 pmol/L; normal range < 13 pmol/L). Blood pressure was adequate for age (95/54 mmHg). Similarly, the diuresis was in the normal range (1.2–2.7 ml/kg/h). With suspicion of a syndrome of inappropriate antidiuretic hormone secretion (SIADH), a fluid restriction was attempted. Although we reported an improvement of sodium concentration (from 126 to 138 mmol/L) at the beginning, the hyponatriemia reappeared over time. We decided to keep Na supplementation at home at the dose of 4 mEq/kg/day. Since she was one year and a half old, her sodium concentrations have been in the normal range, leading us to progressively reduce Na supplementation and to decide a definitive stop at the age of 6. During the follow up, we did not evidence hypertension and renal function persisted normal. Finally, DNA sequencing for AVPR2 mutation was performed, resulting negative.

### Congenital bone abnormalities and mineral metabolism

At the age of one year, she showed mild hypovitaminosis D (25 ng/mL, normal range > 30 ng/mL) with moderately high PTH concentration (100 pg/mL, normal range 15–65 pg/mL). Serum calcium and phosphate were 9.21 mg/dL (normal range for age: 8.7–10.8 mg/dL) and 6.03 mg/dL (normal range for age: 4.0–6.5 mg/dL), respectively. Urinary calcium and phosphate were 12.76 mg/dL and 36.7 mg/dL respectively, urinary calcium/creatinine ratio was 0.85 (normal range for age: 0.03–0.76). Clinically our patient did not show signs of Albright hereditary osteodystrophy (AHO). She started an oral supplementation with cholecalciferol with normalization of PTH and 25-hydroxyvitamin D concentrations. At present, she regularly takes cholecalciferol and presents normal PTH and 25-hydroxyvitamin D levels.

**Fig. 2:**
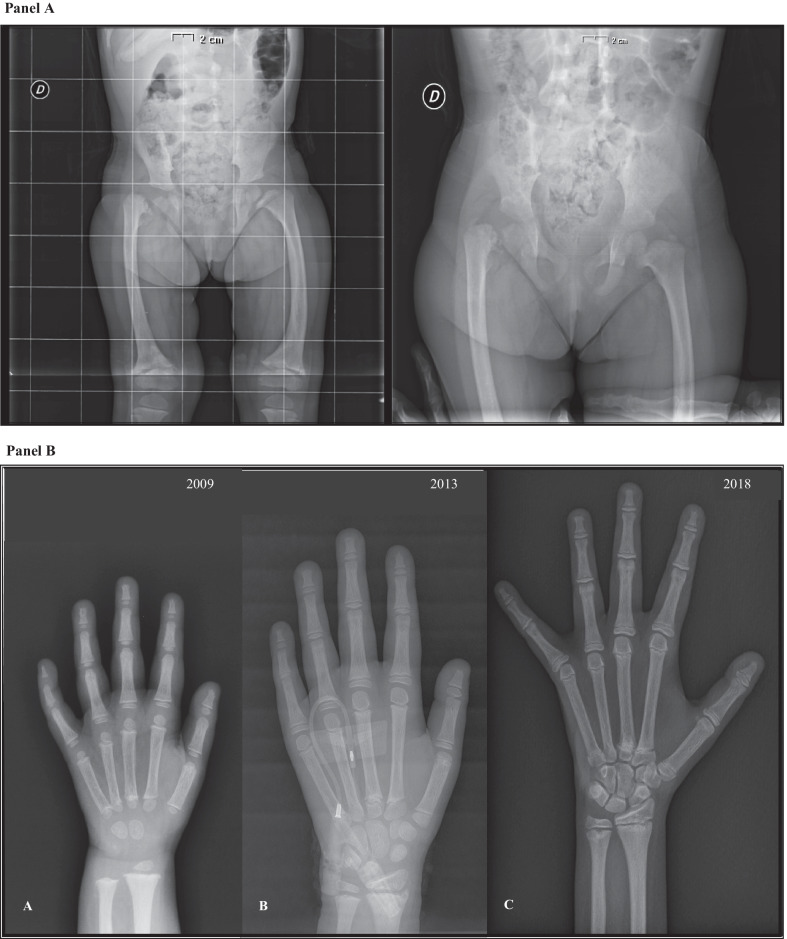
Bone abnormalities. **A** Pelvic X-ray of our patient evidenced right coxa vara. **B** Evolution of bone age through years. The progressive shape change of metacarpals that took a tubular form appears evident

At the age of 2.5 years she presented difficulty walking, we evidenced a dysmetria of lower limbs without other clinical signs and consequently we performed a X-ray of the lower limbs, which showed a congenital right coxa vara (Fig. 2, panel A) and the necessity for surgical intervention of osteostomy. At the age of 6, in consequence to a minor trauma at home, she had a fracture of the femur that needed surgical reduction. On this occasion DXA scan showed a mild reduction of bone mineral density (Z-score: -1.2) and markers of bone turnover were increased (CTX 1.55 ng/mL, normal range for age: 0.20–1.25 ng/mL; osteocalcin 17.7 nmol/L, normal range for age: 3.7–14.9 nmol/L). As far as bone alterations are concerned, she presented metaphyseal irregularity with widening of the growth plate at knees and at wrists’ level. Moreover, she presented a tubular appearance of metacarpals that became more evident over time (Fig. 2, panel B). The last DXA scan at the age of 12 years was normal (femur BMD 1.002 g/cm^2^ [Z-score: 1.5]; column BMD 0.947 g/cm^2^ [Z-score: 0.7]) but markers of bone turnover were still high (CTX 3.21 ng/mL, normal range for age: 0.15–2.29 ng/mL; osteocalcin 25.2 nmol/L, normal range for age and sex: 2.2–20.4 nmol/L).

### Subclinical hyperthyroidism

From the 18^th^ month of life, she began to present a low TSH concentration (0.03–0.17 mIU/L, normal range 0.30–4.50) with normal FT4 concentration (11.1–15.3 ng/L, normal range 8.5–17.5). At the last evaluation, her thyroid functionality confirmed suppressed TSH values with FT4 in the normal range. Thyroid ultrasonography was normal. She has never been subject to any therapy for this problem.

### Precocious thelarche and pubarche

At the age of 4 pubarche (Tanner Ph2) was found. Her adrenal function was normal, but her ACTH level was still unmeasurable. Adrenal glands at abdominal ultrasonography were normal. During the follow up we evidenced a gradual progression of her pubarche (Tanner Ph3) with her androgen levels always in the normal range. Similarly, since she was 7, she has been presenting a thelarche (Tanner T2) with prepubertal gonadotropin’s response to GnRH-analogous stimulation (FSH before: 1.0 U/L, FSH after stimulation: 8.2 U/L; LH before: 0.1 U/L, LH after stimulation: 1.0 U/L). Estradiol concentration was borderline (105 pmol/L, normal range < 130 pmol/L) with no variation after 4 h stimulation, and pelvic ultrasonography excluded the presence of follicles and cysts in ovary. As time passed, the thelarche worsened (Tanner T3), in the presence of normal gonadotropin and estradiol concentrations’ and in the absence of pubertal uterine size at pelvic ultrasonography. Her last bone age was slightly advanced in relation to her chronological age (12 years vs 11 years and 6 months old) (Figs. 1, 2, panel B). At the age of 13 and half she reached menarche. Subsequently, periods were regular.

### Diagnosis

As she presented low levels of ACTH and TSH with normal cortisol and FT4 secretion, premature thelarche and pubarche, congenital bone abnormalities, persistent hyponatremia from birth to 6 years old, we hypothesized a relationship among these heterogeneous hormonal dysfunctions. Since receptors for ACTH, TSH, ADH, PTH, LH and FSH are members of the G protein-coupled receptor superfamily of integral membrane proteins and are coupled to the Gs protein, we investigated the *GNAS* gene, as well as Gs activity in peripheral erythrocytes, identifying the new missense variant p.Ile56Phe in exon 2.

## Methods

The activity of Gsα protein was in vitro analyzed from samples of erythrocyte membranes, recovered from heparinized blood samples. Briefly, after solubilization and activation of the Gs a with guanosine 5’-[g-thio]triphosphate (GTPgS), the generation of cAMP through adenylyl cyclase from turkey red cell membranes was measured in the presence of ATP by RIA (IBL International, Hamburg, Germany). Results in triplicate of index and parents were expressed as a percentage of activity values determined in 10 healthy reference samples (range 85–115%) [18, 19].

The presence of the heterozygous missense c.166A > T—p.(Ile56Phe) *GNAS* variant (nt and pt numbering based on the LRG sequence, *GNAS* mutations database available at: http://databases.lovd.nl/shared/refseq/GNAS_NM_001077488.2_codingDNA.html) was investigated in both the index and parents by direct sequencing of 1–13 *GNAS* coding exons and flanking intronic sequences (ENSEMBL ID: ENSG00000087460), as previously described [20, 21].

The *GNAS* genetic variant discovered was further confirmed by target NGS sequencing using the Illumina MiSeq platform and the HaloPlex Target Enrichment System kit (Agilent Technologies, Santa Clara, CA). Our custom panel included coding regions and exon–intron boundaries regions of the following genes: *GNAS* (NM_001077488), *HDAC4* (NM_006037), *HOXD13* (NM_000523), *PDE3A* (NM_000921), *PDE4D* (NM_001165899), *PRKAR1A* (NM_002734), *PTH1R* (NM_000316), *PTHLH* (NM_198965), *STX16* (NM_001001433), *TRPS1* (NM_014112) e *PRMT7* (NM_019023).

A Metilation Specific Multiplex Ligation-dependent Probe Amplification (MS-MLPA) (SALSA MS-MLPA M031 GNAS—MRC Holland) was performed to exclude the presence of any *GNAS* imprinting defect and/or deletions/duplications.

Finally, western blot analysis was executed. Red cell membrane proteins were analyzed by SDS-PAGE according to Fairbanks et al. [22] with minor modifications [23]. Protein fractions (30 µg) were separated on 8.5% SDS/polyacrylamide gels and transferred to a nitrocellulose filter. Blots were blocked overnight at 4 °C with 10% powdered skimmed milk in TBTS. Anti-Gαs mouse monoclonal antibody (Santa Cruz Biotechnology, Dallas, TX, USA) was diluted in TBST buffer with 2.5% milk and was used at 1:200 for 2 h at room temperature. Secondary antibody was diluted in TBST buffer with 2.5% milk 1:2000 and incubated for 1 h at room temperature. Anti-GAPDH antibody (Ambion, Life Techonologies, ThermoFisher, CA, USA) was used at 1:4000 for 1 h at room temperature. Chemiluminescence was detected using ChemiDOC-IT Imaging System (UVP, Upland, CA) and analyzed with NIH ImageJ software.

The genetic variant found in the patient was described according to the reference sequence covering the GNAS transcript NM_001077488.1 from the human assembly GRCh37 (also known as hg19).

### Ethics approval

The study was conducted in compliance with the terms of the Helsinki II Declaration and written informed consent for the enrolment and for the publication of individual clinical details was obtained from parents. In our country, namely Italy, this type of clinical study does not require Institutional Review Board/Institutional Ethics Committee approval to publish the results.

The authors have obtained consent to publish from the parent of the children.

## Results

**Fig. 3:**
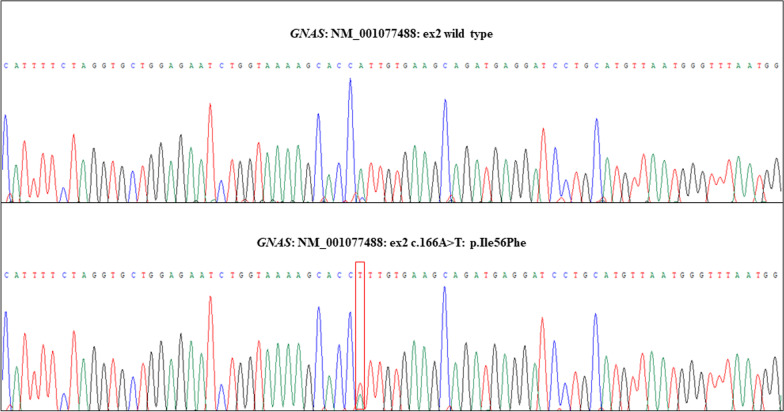
Electropherogram of the novel *GNAS* variant by Sanger sequencing

**Fig. 4:**
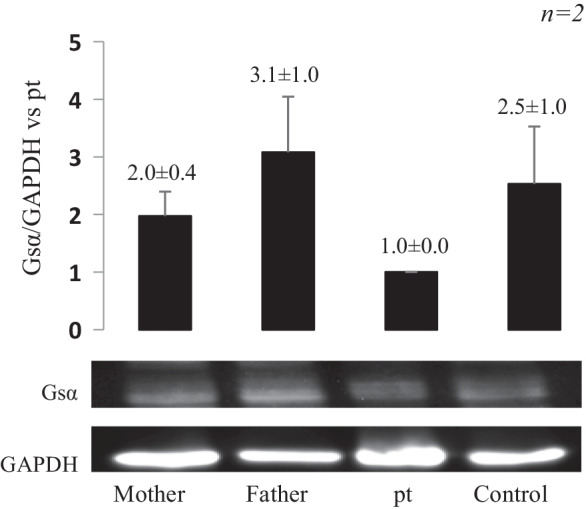
Analysis of Gsα protein expression levels. Representative immunoblot and densitometrical analysis showing Gsα expression among the Family members. Red cell membrane protein exctracts were obtained from all samples, and two separate Western blot experiments were performed (n = 2). GAPDH was used as housekeeping gene for normalization. Results represent mean ± standard deviation (SD) and are expressed as fold-increase vs proband. No statistically significant difference was found between samples. (*Pt* = *patient*)

*GNAS* genetic analysis identified a new missense variant p.Ile56Phe in exon 2, inherited from the mother (Fig. 3). This variant likely affects Gsα protein function, as demonstrated by a reduced activity of 50%. The analysis of her parents showed a 20–25% reduction in Gsα protein activity in the mother and a normal function in the father. Anyhow, her mother has always been asymptomatic, with a thyroid function in normal range. Accordingly, western blot analysis of Gsα expression in red cell membrane proteins revealed lower levels of Gsα protein in the patient with respect to her mother, father and grandmother (Fig. 4).

This variant was absent from public databases such as ClinVar, HGMD and LOVD, and in silico predictions assessed a pathogenetic effect (Table 1). A frameshift mutation (p.I56Rfs*2), affecting the same aminoacid found substituted in our patient, was previously reported in the LOVD *GNAS* database (The RefSeq used as LRT in the *GNAS* LOVD database is NM_001077488). This information, together with in silico predictions and the reduced functional activity of Gsα in eritrocytes of the patient, further support the likely pathogenetic effect of the p.I56F variant.

**Table 1:** Results of the in silico prediction analysis of pathogenicity of the novel *GNAS* variant

Germline allelic variant	Pathogenicity Prediction/Score						Conservation score					
	Varsome (Genome Interpretor)*	ACMG/AMP* criteria	DANN^(a)^	Mutation Taster^(b)^	FATHMM-MKL^(c)^	EIGEN^(d)^	GERP^(e)^	PhyloP100way^(f)^	PhastCons100way^(g)^	fitCons (GM12878/ H1-hESC/ HUVEC)^(h)^	Integrated fitCons^(i)^	bstatistic^(l)^
NM_0011077488: c.166A > T p.I56F	Likely pathogenic	PM1^#1^ moderate PM2^#2^ moderate PP2^#3^ supporting PP3^#4^ supporting	0.99	Disease causing	Damaging	Pathogenic	5,63	8.95	1	0.67/0,68/0,72	0.65	986

^*^The American College of Medical Genetics and Genomics (ACMG)/Association for Molecular Pathology (AMP) criteria: #1 Null variant (nonsense, frameshift, canonical ± 1 or 2 splice sites, initiation codon, single or multiexon deletion) in a gene where LOF is a known mechanism of disease—#2 Absent from controls (or at extremely low frequency if recessive) in Exome Sequencing Project, 1000 Genomes Project, or Exome Aggregation Consortium—#3 Multiple lines of computational evidence support a deleterious effect on the gene or gene product (conservation, evolutionary, splicing impact, etc.)—#4 Pathogenic computational verdict based on 13 pathogenic predictions vs no benign predictions; (a) pathogenicity scoring methodology based on deep neural networks; (b) the pathogenicity of a variant based on evolutionary conservation, splice-site, mRNA, protein and regulatory features; (c) predict the functional consequences of non-coding and coding single nucleotide variants (SNVs); (d) spectral approach to the functional annotation of genetic variants in coding and noncoding regions; (e) Genomic Evolutionary Rate Profiling, a conservation score calculated by quantifying substitution deficits across multiple alignments of orthologues using the genomes of 35 mammals; (f) Phylogenetic P-values, scores based on multiple alignments of 99 vertebrate genome sequences to the human genome; (g) identify evolutionarily conserved elements in a multiple alignment, given a phylogenetic tree, based on 100 vertebrate genomes (including human); (h) FITness CONSequences of functional annotation, identifies genomic regions under selective pressure by integrating epigenomic signals from three ENCODE cell lines (GM12878, H1-hESC and HUVEC) with selective pressure inferred using the INSIGHT ((Inference of Natural Selection from Interspersed Genomically coHerent elemenTs) method; (i) integrates functional assays (such as ChIP-Seq) with selective pressure inferred using the INSIGHT method.; (l) indicates the expected fraction of neutral diversity that is present at a site, based on human single nucleotide polymorphism (SNP) data

**Fig. 5:**
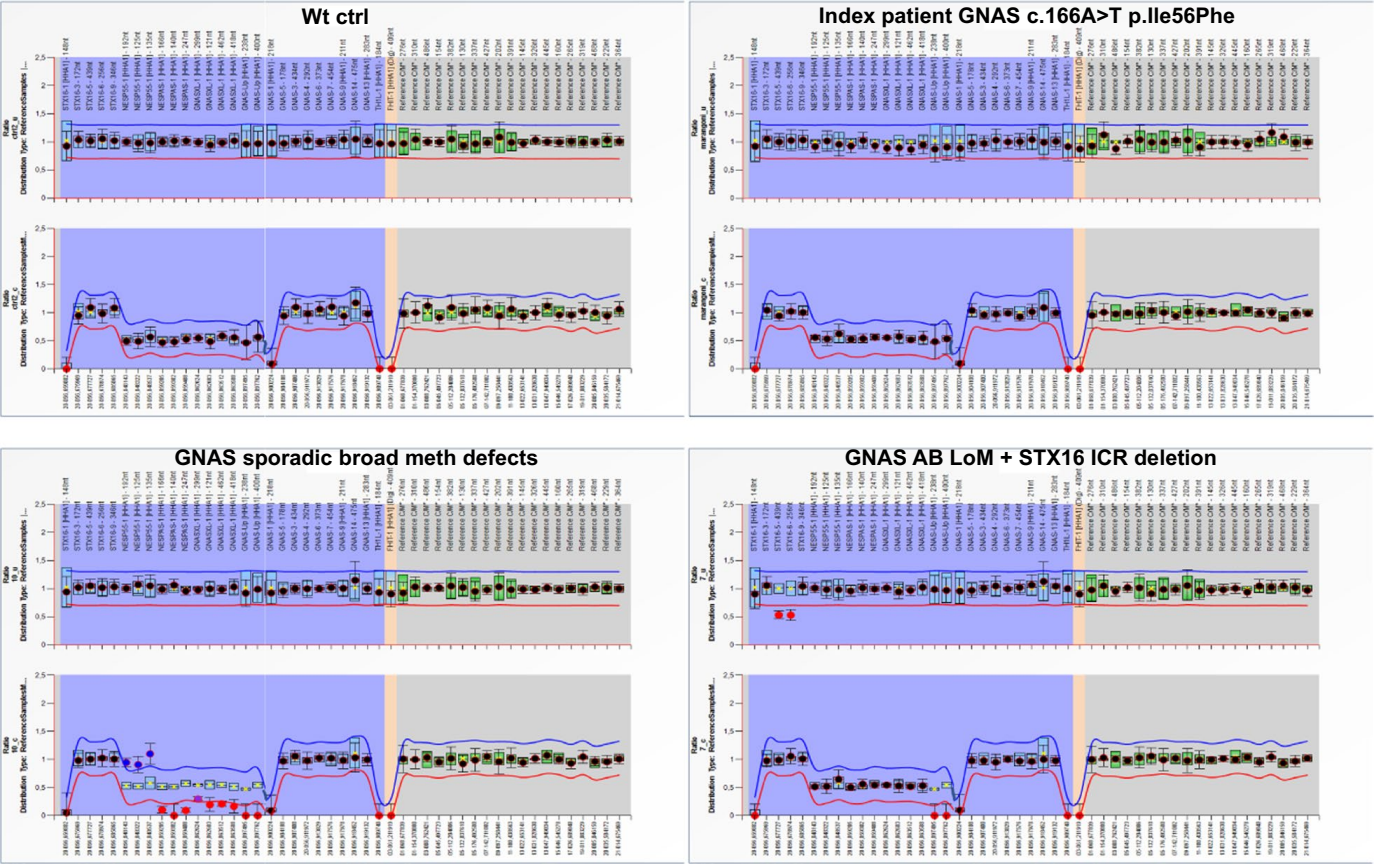
GNAS locus copy number and methylation status analysis by MS-MLPA

No additional potentially causative genetic variants were identified in our patient (Table 2). Finally, we excluded the presence of any *GNAS* imprinting defect, both autosomal dominant and sporadic, and/or deletions/duplications (Fig. 5).

**Table 2:** Exonic nonsynonymous variants identified by NGS target sequecing

Chr	Start	End	Ref	Alt	dbSNP	Otherinfo	Func.refgene	Gene.refgene	ExonicFunc.refgene	AAChange.refgene	1000G_ALL	1000G_AFR	1000G_AMR	1000G_EAS	1000G_EUR	1000G_SAS	In silico/db prediction
chr12	20522252	20522252	G	A	rs12305038		exonic	PDE3A	nonsyn SNV	PDE3A:NM_000921: exon1:c.G34A:p.D12N	0.33	0.51	0.22	0.31	0.31	0.24	Benign
chr20	57244396	57244396	G	A	rs41276950		exonic	STX16	nonsyn SNV	STX16:NM_001134772: exon4:c.G431A:p.R144Q NM_003763: exon4:c.G380A:p.R127Q	0.014	0.0023	0.016		0.054	0.0041	Benign
chr20	57470693	57470693	A	T		het	exonic	GNAS	nonsyn SNV	GNAS:NM_001077488: exon2:c.A166T:p.I56F							Novel:pathogenetic

## Discussion

We report the case of a girl who presented the heterozygous missense variant c.166A > T in exon 2 of the *GNAS* gene. Such a variant resides on maternal allele, with a consequent reduction of 50% in Gsα protein activity. The infant displayed a characteristic combination of clinical and laboratory data, including persistent hyponatremia from birth to the age of 6, congenital bone abnormalities, low levels of ACTH and TSH with normal cortisol and FT4 secretion, premature thelarche and pubarche. Although the variant is associated to a reduced activity of the function of Gsα protein, this unusual phenotype suggests a mild functional gain. Therefore, these different and apparent unrelated symptoms might be part of a more complex previous unrecognized multisystem disorder.

The severe but asymptomatic infantile hyponatremia was primarily attributed to CAH and treated for some months with cortisone acetate but the absence of concomitant hyperkalaemia and virilising clinical signs led us to change the first diagnostic hypothesis. Later, hyponatremia was associated to a nephrogenic syndrome of inappropriate antidiuresis (NSIAD), a rare condition caused by impaired renal capacity to excrete a free water load into the urine under undetectable or very low plasma ADH levels. However, in our patient ADH concentrations were always in the normal range, not supporting the NSIAD hypothesis, which was further dismissed after genetic analysis of AVPR2 gene had resulted negative. Only after the finding of the missense variant p.Ile56Phe in exon 2 of *GNAS* gene we related these data to the hyponatremia. In fact, we postulated that this variant had led to NSIAD by an agonist-independent activation of the vasopressin 2 receptor regulated pathway. In this context, it is not easy to understand why our patient maintained a persistent normal level of ADH in presence of blood hypo-osmolality, hyponatriemia and inappropriately high urine osmolality. It is probably due to the methodology used in 2007 for the ADH determination that, at that time, was not performed in our center. In any case, these data made more complicated for us to understand her clinical condition on the basis of her hyponatremia. In fact only in recent years, some clinical conditions similar to ours have been described [14, 15, 24–26]. According to the hypothesis of the authors of one of these papers, the resolution of hyponatremia after early infancy was probably due to adaptive mechanisms, such as changes in nutrition and self-regulation of thirst-driven fluid intake [24]. A fluid restriction was performed in our patient, with a partial resolution of the hyponatriemia. The persistence of occasional asymptomatic hyponatremia, associated to the difficulty experienced in maintaining a fluid restriction during the first months of life, induced us, probably incorrectly, to continue sodium supplementation in early infancy. Only as time passed, consequent changes in feeding and a spontaneous reduction of fluid intake led to the normalization of sodium concentrations. However, it seems possible that an excessive free water intake may lead to hyponatremia again in the future.

In detail, in 2016 Wentworth et al. reported a novel heterozygous missense mutation of *GNAS* (c.163A < G, p.T55A) in a girl presenting with neonatal SIADH, osteolysis and bone fragility with multiple fracture. This variant was associated in vitro with a decreased cAMP production, consistent with loss of Gsα activity [26]. On the contrary, *Biebermann* and colleagues described a new *GNAS* variant (c.1136 T > G; p.F376V in exon 13) in two unrelated patients presenting a combination of clinical signs similar to those presented by our patient, namely severe asymptomatic hyponatremia in infancy (similar to NSIAD), skeletal and growth plate abnormalities, GnRH-independent precocious puberty and apparent PTH resistance in the proximal but not distal renal tubules. This heterozygous de novo variant was found on the maternal *GNAS* allele and presented clinical findings suggesting both gain and loss of Gsα function. The explanation for this new data lies in an increased G protein activity at basal rather than stimulated state, implying a different ligand-dependent protein conformation and subsequently an altered signaling cascade [14]. More recently, *Miyado* and collaborators have published a report regarding two families with NSIAD, in which the whole-genome sequencing revealed two novel germline-derived *GNAS*-Gsα variant p.(F68_G70del) in exon 2 and p.(M255V) in exon 10, both on maternal allele [25]. Also, the novel *GNAS* variant found in our patient was of maternal origin, therefore we believe that this might be a relevant factor in the genesis of this multisystem disorder. Recent research has suggested that the effects of these variants of *GNAS* gene are exhibited in a tissue-specific manner [14, 24–26]. The phenotypical and biochemical variability could be consequently referred to the differential tissue-specific imprinting of *GNAS* gene, with predominant or exclusive maternal expression. In particular, *GNAS*-Gsα is predominantly expressed from the maternal allele in renal proximal tubule, thyroid, gonads, pituitary and the paraventricular nucleus of the hypothalamus [4, 27–30]. Furthermore, a higher maternal expression of Gsα have been proved in human lymphoblasts, peripheral blood mononuclear cells, mammary adipose tissue and heart [31]. Unfortunately, in our patient no tissue-specific analysis of Gα activity has been conducted, which could clarify the effect of this new variant on each tissue. However, the maternal origin of this variant would be coincidental for the development of NSIAD and of subclinical hyperthyroidism in our patient.

Reduced Gsα activity is usually related to inactivating mutations of *GNAS* gene, which are involved in development of different phenotypic expression that varies from PHP to PPHP to POH [3, 12, 13]. In particular, PHP and PPHP include variable signs of AHO, which is characterized by a mixture of features including round face, short stature, brachydactyly, subcutaneous ossification, and intellectual disability [3, 4]. Despite expectations of a reduced Gsα activity, our patient did not show any sign of resistance to hormone activity. She does not present short stature, brachydactyly, brachymetacarpia, and intellectual disability, according the clinical characteristic of patient described by *Wentworth* [26]. On the contrary, Gsα activating variants result in constitutive cAMP signaling that leads to different clinical phenotypes such as fibrous dysplasia or MAS [3, 16, 17]. Hyperthyroidism, Cushing syndrome, and pituitary gigantism/acromegaly could also be part of the clinical presentation of MAS if mutated cells are present in those tissues [3, 12]. Our patient does not display fibrous dysplasia although she shows a certain degree of bone fragility and a coxa vara and presents increased levels of bone-turnover markers which could resemble a constitutive activation of Gsα at bone level. Unfortunately, no histological data are available on bone sample. Whereas our patient does not present café-au-lait skin pigmentation and Cushing syndrome, she evidences premature thelarche and pubarche and subclinical hyperthyroidism, which are also consistent with a gain-of-function effects. Bone age was advanced in the first years of life and now it corresponds to chronological age; she has already reached a height appropriate to her familiar target despite not having completed the pubertal development.

It is worth emphasizing that, although the variant found in our child induces a 50% reduction of *GNAS* activity, most of the clinical signs that she presents suggest a gain of Gsα function. The cause for this apparent discrepancy remains unknown.

Finally, all symptoms presented by our patient were mostly mild. This is consistent with the fact that *GNAS* expression is finely regulated at many levels and that some transduction signals are nonresponsive to the mild gain of function effects. However, future studies are needed in order to clarify the underlying mechanisms.

As above mentioned, the major limitations of this report are the lack of tissue-specific analysis and the absence of a functional analysis of the new discovered variant. Despite these limitations, we describe for the first time a specific phenotype and relate the clinical symptoms to a new variant. Moreover, the genetic analysis excluded other alterations in genes known to be involved in PHP and PHP related phenotypes. Furthermore, this young girl will be followed up in case of new phenotypical or biochemical appearances.

## Conclusions

In summary, the new variant c.166A > T (p.Ile56Phe) on exon 2 of *GNAS* gene, originated on maternal allele, has been described in a young girl as likely causative of a new multisystemic disorder characterized by hyponatremia, subclinical hyperthyroidism, subclinical hypercortisolism, precocious thelarche and pubarche and congenital bone abnormalities. Different complex mechanisms (parental imprinting, somatic mosaicism, tissue-specific expression, genetic regulation) might be involved in the development of these phenotypical and biochemical features. Knowing of the possible effects of this variant might be helpful in the clinical management of these patients and in the development of specific treatment.

## Data Availability

Data sharing not applicable to this article as no datasets were generated or analysed during the current study.
