# Correction to: A complex pheotype in a girl with a novel heterozygous missense variant (p.Ile56Phe) of the GNAS gene

**DOI:** 10.1186/s13023-022-02316-7

**Published:** 2022-04-19

**Authors:** Paolo Cavarzere, Andrea Gastaldi, Francesca Marta Elli, Rossella Gaudino, Erika Peverelli, Milena Brugnara, Susanne Thiele, Francesca Granata, Giovanna Mantovani, Franco Antoniazzi

**Affiliations:** 1 Pediatric Division, Department of Pediatrics, University Hospital of Verona, Piazzale Stefani 1, 37126 Verona, Italy; 2 Endocrinology Unit, Department of Clinical Sciences and Community Health, University of Milan, Fondazione IRCCS Ca’ Granda Ospedale Maggiore Policlinico, Milan, Italy; 3 Pediatric Clinic, Department Surgical Sciences, Dentistry, Gynecology and Pediatrics, University of Verona, Verona, Italy; 4 Division of Paediatric Endocrinology and Diabetes, Department of Pediatrics, University of Lübeck, Luebeck, Germany; 5 General Medicine Unit, Fondazione IRCCS Ca’ Granda Ospedale Maggiore Policlinico, Milan, Italy; 6 Regional Center for the Diagnosis and Treatment of Children and Adolescents Rare Skeletal Disorders, Pediatric Clinic, Department of Surgical Sciences, Dentistry, Gynecology and Pediatrics, University of Verona, Verona, Italy

## Correction to: Orphanet Journal of Rare Diseases (2022) 17:83 10.1186/s13023-022-02252-6

Following the publication of the original article [1] we were informed that several of the co-authors’ given and family names had unfortunately been interchanged.

The correct author names are shown here below:

Paolo Cavarzere, Andrea Gastaldi, Francesca Marta Elli, Rossella Gaudino, Erika Peverelli, Milena Brugnara, Susanne Thiele, Francesca Granata, Giovanna Mantovani and Franco Antoniazzi

The author names have been corrected in the author list of this Correction and updated in the original article.
